# CCR5 knockout suppresses experimental autoimmune encephalomyelitis in C57BL/6 mice

**DOI:** 10.18632/oncotarget.8097

**Published:** 2016-03-15

**Authors:** Sun Mi Gu, Mi Hee Park, Hyung Mun Yun, Sang Bae Han, Ki Wan Oh, Dong Ju Son, Jae Suk Yun, Jin Tae Hong

**Affiliations:** ^1^ College of Pharmacy and Medical Research Center, Chungbuk National University, Osongsaengmyeong 1-ro, Osong-eup, Heungdeok-gu, Cheongju-si, Chungcheongbuk-do, Republic of Korea; ^2^ Department of Maxillofacial Tissue Regeneration, School of Dentistry and Research Center for Tooth and Periodontal Regeneration (MRC), Kyung Hee University, Kyungheedae-ro, Dongdaemun-gu, Seoul, Republic of Korea; ^3^ Pharmacological Research Division, National Institute of Food and Drug Safety Evaluation (NIFDS), Ministry of Food and Drug Safety (MFDS), Osongsaengmyeong 2-ro, Osong-eup, Heungdeok-gu, Cheongju-si, Chungcheongbuk-do, Republic of Korea

**Keywords:** multiple sclerosis, C-C chemokine receptor 5, cytokine, chemokine, experimental autoimmune encephalomyelitis, Immunology and Microbiology Section, Immune response, Immunity

## Abstract

Multiple sclerosis (MS) is an inflammatory disease in which myelin in the spinal cord is damaged. C-C chemokine receptor type 5 (CCR5) is implicated in immune cell migration and cytokine release in central nervous system (CNS). We investigated whether CCR5 plays a role in MS progression using a murine model, experimental autoimmune encephalomyelitis (EAE), in CCR5 deficient (CCR5^−/−^) mice. CCR5^−/−^ and CCR5^+/+^ (wild-type) mice were immunized with myelin oligodendrocyte glycoprotein 35-55 (MOG_35-55_) followed by pertussis toxin, after which EAE paralysis was scored for 28 days. We found that clinical scoring and EAE neuropathology were lower in CCR5^−/−^ mice than CCR5^+/+^ mice. Immune cells (CD3^+^, CD4^+^, CD8^+^, B cell, NK cell and macrophages) infiltration and astrocytes/microglial activation were attenuated in CCR5^−/−^ mice. Moreover, levels of IL-1β, TNF-α, IFN-γ and MCP-1 cytokine levels were decreased in CCR5^−/−^ mice spinal cord. Myelin basic protein (MBP) and CNPase were increased while NG2 and O4 were decreased in CCR5^−/−^ mice, indicating that demyelination was suppressed by CCR5 gene deletion. These findings suggest that CCR5 is likely participating in demyelination in the spinal cord the MS development, and that it could serve as an effective therapeutic target for the treatment of MS.

## INTRODUCTION

The autoimmune response in the central nervous system (CNS) plays a critical role in chronic inflammation and demyelination in human multiple sclerosis (MS) and in the animal model, experimental autoimmune encephalomyelitis (EAE) [1]. EAE is widely used as and animal model of human CNS demyelinating diseases, including MS [2]. It is mediated by activated myelin antigen-specific CD4^+^ Th1 cells and is characterized histologically by Ag-specific and nonspecific CD4^+^ and CD8^+^ Th1 cell infiltration [3]. Oligodendrocyte damage and apoptosis in response to CNS autoimmune inflammation are widely considered to be the pathological bases of MS and EAE, and are believed to result from the release of pro-inflammatory mediators and nitric oxide (NO) by activated T cells, macrophages, B cells, NK cells and activated glial cells [4].

In patients, each subsequent EAE episode following initial disease onset has been correlated with increased expression of inflammatory cytokines, such as TNF-α and IFN-γ [5, 6]. Initial reports claimed that *in vivo* TNF blockade in mice and rats resulted in EAE amelioration [7, 8]. Further studies using TNF-deficient mice identified TNF as critical pathogenic cytokine that induces chemokine expression in the CNS [9, 10]. IFN-γ aggravates the course of neuroinflammatory disorders through microglia activation [11]. Interleukin-1 (IL-1) is produced by a variety of cells, such as monocytes/macrophages, epithelial and endothelial cells and glial cells [12]. These cytokine plays a crucial role in leukocyte extravasation into inflammatory sites through upregulation of intracellular adhesion molecule-1 and vascular cell adhesion molecule-1 [13]. Monocyte chemotactic protein 1 (MCP-1) is unregulated T_h_1 immune responses during the acute phase of disease induced by myelin oligodendrocyte glycoprotein 35-55 (MOG_35-55_) or other encephalitogenic antigens [14-16]. Vaccination with naked MCP-1 DNA inhibited EAE in mice [17]. Our previous study also found that MCP-1 levels were significantly reduced in lung tumor tissue and blood in CCR5-deficient mice compared with controls [18].

Chemokines interact with their respective cell surface receptors and mediate the recruitment of specific leukocyte subpopulations to sites of inflammation. Disruption of C-C chemokine receptors (CCR) may lead to impaired monocyte function, including chemokine-directed chemotaxis, and CCR manipulation may aid in inflammatory disease resistance [19, 20]. C-C chemokine receptor 5 (CCR5) is implicated in immune cell migration and cytokine release in the CNS, and may play a role in the pathogenesis of MS and EAE. CXCL12 is associated infiltrating leukocytes, CXCR7 antagonist inhibited function of CXCL12, so CXCR7, a CXCL12 receptor), impacted both MS severity and recovery during EAE [21], however, CXCR6 is required for T cell infiltration into spinal cord following cortical injury [22]. *In vivo* studies indicated the MCP-1 and CCR2 might promote EAE initiation and progression. In addition, CCR1, 3 and 5 antagonist prevented demyelination syndromes [23]. EAE-mediated central nerve damage was ameliorated in IL-9 deficient mice *via* decreased CCR2, CCR5 and CCR6 expression [24].

Immunologic processes contribute to the initiation and continuation of MS and EAE, and recent studies have indicated that microglia, astrocytes and infiltrating immune cells have separate roles in MS lesion development [25]. The role of cytokines as important regulatory elements in these immune processes has been well established in EAE and the presence of cytokines in cells at the edges of MS lesions has been observed [26]. To assess the importance of CCR5 signaling in EAE pathogenesis, we induced disease in CCR5^−/−^ and CCR5^+/+^ animals using the encephalitogenic peptide, MOG_35-55_. Mice lacking CCR5 were resistant to EAE, failed to develop mononuclear cell infiltrates, and displayed decreased pro-inflammatory responses in the CNS and spinal cord.

## RESULTS

### CCR5 **deficiency** suppressed MOG_35-55_-induced EAE

**Figure 1:**
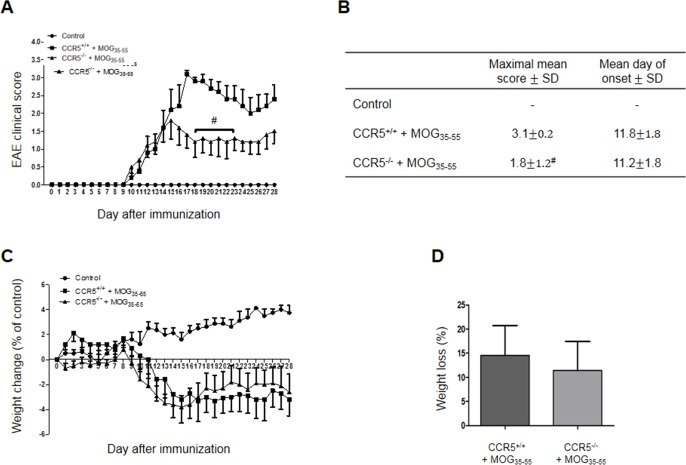
MOG_35-55_-induced EAE in CCR5^+/+^ and CCR5^+/+^ mice Mean clinical scores of age- and sex-matched control mice after saline and CCR5^+/+^ and CCR5^−/−^ mice after induction of EAE with MOG_35-55_ **A.** Control mice group had no symptoms **B.** Maximal mean score was 1.8±0.2 in MOG_35-55_-immunized CCR5^−/−^ mice and 3.1±1.2 in MOG_35-55_-immunized CCR5^+/+^ mice. Mean day of onset was the same for CCR5^+/+^ (11.8±1.8) and CCR5^−/−^ (11.2±1.8) mice. Mean body weight (g) was compared to day 0 in each group after injection of saline or MOG_35-55_ for 28 days **C.** At day 28, control mouse body weight had increased 3.8±2.2 g, but had decreased 3.2±3.2 g and 2.6±3.0 g in MOG35-55-immunized CCR5^+/+^ and CCR5^−/−^ mice, respectively. Weight loss (%) was not different between MOG_35-55_-induced CCR5^+/+^ (14.6±13.8) and CCR5^−/−^ (11.4±13.4) mice **D.** Values are presented as mean ± SEM. ^#^*p* < 0.05 compared to MOG_35-55_-immunized CCR5^+/+^ mice.

CCR5^+/+^ and CCR5^−/−^ mice sensitized at 8 weeks of age developed clinical signs of MOG_35-55_ peptide-induced EAE. Paralysis first appeared at day 9 in both groups. CCR5^−/−^ mice exhibited symptoms faster CCR5^+/+^ mice at the initiatory stage of paralysis, but mean clinical score was lower for the CCR5^−/−^ group (Figure 1A and 1B). Mean body weight changes were similar between CCR5^+/+^ and CCR5^−/−^ mice (Figure 1C and 1D).

### Spinal cord injuries were decreased in CCR5^−/−^ mice

**Figure 2:**
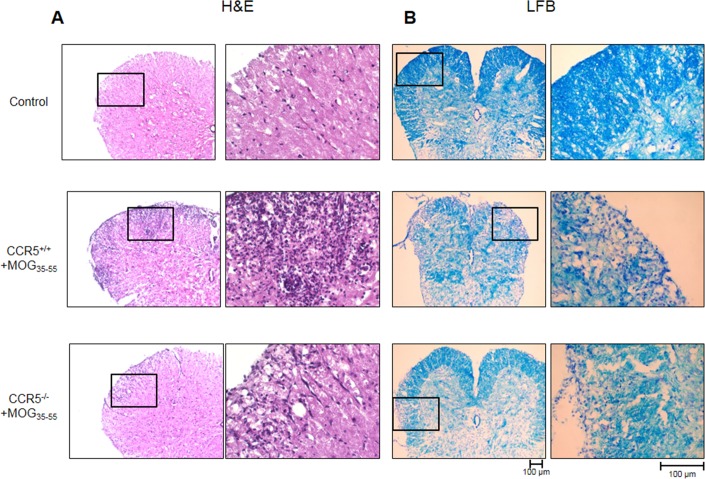
H&E (cell infiltration) and LFB (demyelination) staining was performed in 16 μm-thick mouse spinal cord sections Cell infiltration **A.** and demyelination **B.** was greater in MOG_35-55_-immunized CCR5^+/+^ mice than in MOG_35-55_-immunized CCR5^−/−^ mice.

Hematoxylin and Eosin (H&E) staining in spinal cord sections showed that mononuclear cell infiltration into the injured area decreased in CCR5^−/−^ as compared to CCR5^+/+^ mice (Figure 2A). Similarly, CCR5^−/−^ mouse spinal cord sections showed less reduction in Luxol Fast Blue (LFB) staining as compared to those of CCR5^+/+^ mice, indicating that less demyelination occurred in CCR5^−/−^ mouse spinal cords (Figure 2B).

### Inflammatory cytokine levels were decreased in CCR5^−/−^ mice

**Figure 3:**
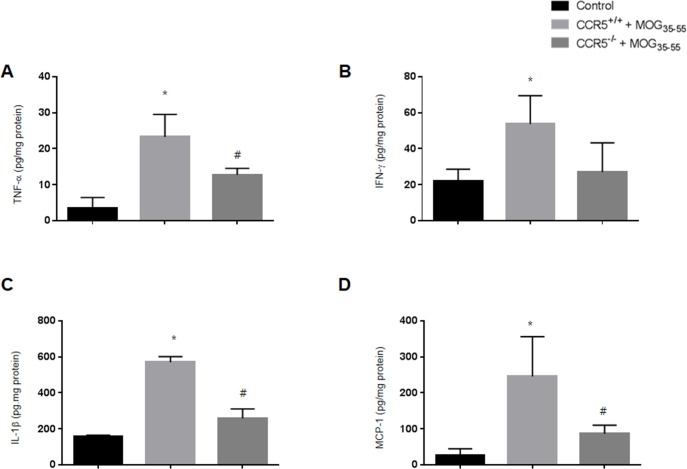
Levels of pro-inflammatory cytokines (TNF-α, IFN-γ and IL-1β) and chemokines (MCP-1) in mouse spinal cords were measured by ELISA. A TNF-α protein expression in MOG_35-55_-immunized CCR5^+/+^ and CCR5^−/−^ mouse spinal cords **B.** IFN-γ expression was not significantly different between MOG_35-55_-immunized CCR5^+/+^ and CCR5^−/−^ mouse spinal cords **C.** MOG_35-55_ injection increased IL-1β expression in CCR5^+/+^ mice compared to CCR5^−/−^ mice **D.** MCP-1 expression in MOG_35-55_-immunized CCR5^+/+^ mice was higher than in CCE5^−/−^ mice. Values are presented as mean ± SEM. ^*^p < 0.05 compared to control, ^#^*p* < 0.05 compared to MOG_35-55_-immunized CCR5^+/+^ mice.

We measured cytokine levels related to EAE *via* ELISA using spinal cord tissue from control, CCR5^+/+^ and CCR5^−/−^ mice. Levels of inflammatory cytokines such as TNF-α and IFN-γ were increased overall in both CCR5^+/+^ and CCR5^−/−^ mice, but were higher in CCR5+/+ mice (Figure 3A and 3B). IL-1β and MCP-1 levels were also was increased in CCR5+/+ mice (Figure 3C and 3D). IL-1β contributes to the activation of auto-antigen-specific immune cells, and MCP-1 was upregulated in the CNS of acute EAE cases.

### Immune cell infiltration was decreased in CCR5^−/−^ mice

**Figure 4:**
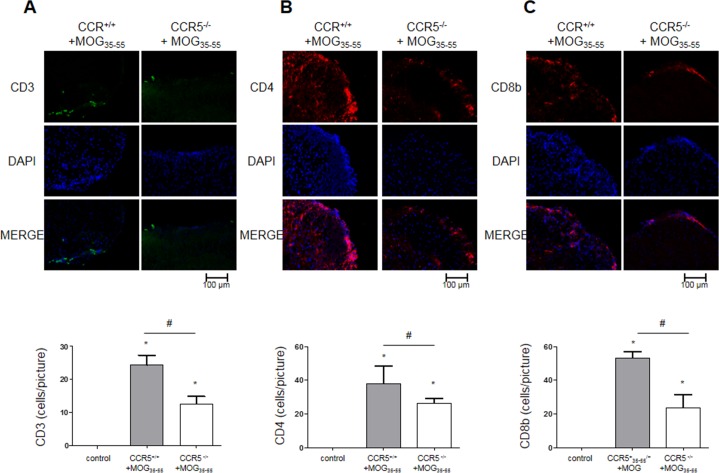
T cell infiltration as shown by IF staining in 16 μm-thick mouse spinal cord sections CD3^+^, CD4^+^, CD8^+^ T cells did not infiltrate control mouse spinal cord tissue. MOG_35-55_-induced mouse spinal cord tissues showed infiltrating CD3^+^, CD4^+^, CD8^+^ T cells **A.**-**C.** Values are presented as mean ± SEM. (*n* = 3) ^*^p < 0.05 compared to control, ^#^*p* < 0.05 compared to MOG_35-55_-immunized CCR5^+/+^ mice.

We detected CD3^+^ (a T cell marker), CD4^+^ (a helper T cell marker), CD8b^+^ (a cytotoxic T cell marker), CD11b^+^ (a macrophage and microglial cell marker), F4/80^+^ (a macrophage marker), CD16+ (an NK cell marker) and CD19^+^ (a B cell marker) *via* immunofluorescence (IF) staining of the spinal cord sections. We observed increased numbers of immune cells (T cells, helper T cells, cytotoxic T cells, macrophages, NK cells and B cells) in the spinal cords of CCR5^+/+^ mice (Figure 4A & S1). These results may explain why CCR5^+/+^ mice had higher clinical scores than CCR5^−/−^ mice.

### Increased myelin protein and decreased oligodendrocytes progenitor cell marker levels in CCR5^−/−^ mice

**Figure 5:**
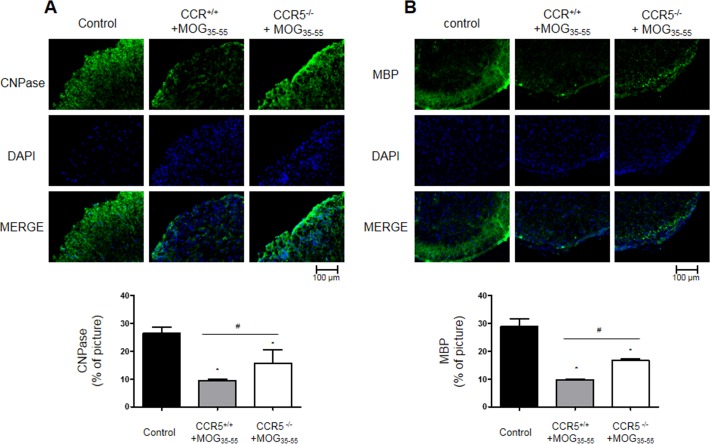
Myelin protein expression as shown by IF staining in 16 μm-thick mouse spinal cord sections CNPase (myelinating oligodendrocyte marker) **A.** and NG2 (oligodendrocyte precursor maker) **B.** expression decreased in MOG_35-55_-induced CCR5^+/+^ mouse spinal cords as compared to CCR5^−/−^ mice spinal cord. Values are presented as mean ± SEM. (*n* = 3) ^*^p < 0.05 compared to control, ^#^*p* < 0.05 compared to MOG_35-55_-immunized CCR5^+/+^ mice.

We used IF staining to detect expression of CNPase (myelinating oligodendrocytes marker), myelin basic protein (MBP), NG2 (a marker of oligodendrocyte progenitor cells; OPCs), O4 (an oligodendrocyte marker), GFAP (a marker of astrocyte activation) and IBA-1 (a marker of microglia cell activation). CNPase and MBP were increased in CCR5^+/+^ mice, while NG2 and O4 were decreased in CCR5^−/−^ mice (Figure 5, S2A & S2B). GFAP and IBA-1 were decreased in CCR5^−/−^ mice as compared to CCR5^+/+^ mice (Figure S2C & S2D).

### Decrease T cell proliferation and inflammatory cytokine levels in Jurkat cells treated with con A and DAPTA

**Figure 6:**
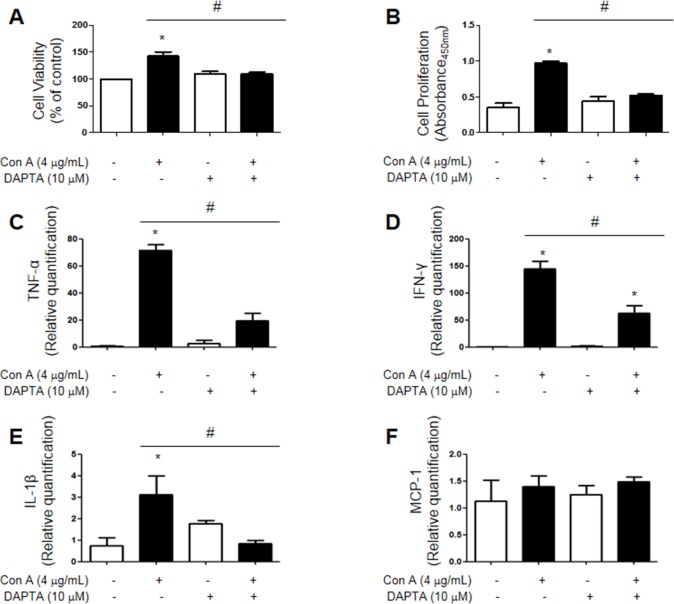
Jurkat cells were stimulated using con A (4 μg/mL) and CCR5 was inhibited using DAPTA (10 μM) Con A treatment enhanced cell viability (MTT assay) **A.** and proliferation (BrdU assay) **B.** compared to controls, but this effect was reversed by DAPTA. As measured by real-time PCR, TNF-α, IFN-γ, IL-1β and MCP-1 levels were increased in con A-treated cells, but were decreased in cells treated with both con A and DAPTA **C.**-**F.** Values are presented as mean ± SEM. (*n* = 3) ^*^p < 0.05 compared to control, #*p* < 0.05 compared to con A-treated cells.

We stimulated Jurkat cells using con A (4 μg/mL) and used a CCR5 antagonist (DAPTA, 10 μM) to suppress CCR5 activity. Cell viability, as measured by MTT assay, was increased by con A treatment and this effect was attenuated by DAPTA treatment (Figure 6A). Cell proliferation, as measured by BrdU assay, showed the same results as cell viability tests (Figure 6B). Jurkat cells were then stimulated under the same treatment conditions and inflammatory cytokine levels were measured using real-time PCR. TNF-α and IFN-γ mRNA levels were increased in con A-treated Jurkat cells, but decreased in cells treated with DAPTA and con A together. IL-1β and MCP-1 mRNAs were rarely detected in all groups (Figure 6C-6F).

## DISCUSSION

Chemokine receptors (CCRs) are involved in the pathogenesis of EAE and autoimmune disease [27]. CCR1^−/−^, CCR2^−/−^, CCR8^−/−^ and CXCR2^−/−^ mice exhibited reduced EAE symptoms compared to controls [9, 28-30]. CCR7^−/−^ mice did not develop EAE [31]. CXCR3 blockade inhibited leukocyte infiltration in the CNS [32]. CCR6^−/−^ mice showed delayed EAE onset and the course of disease was more severe in CCR6^+/+^ mice [33]. However critical mechanisms and roles of CCR5 in EAE pathogenesis are not clear yet.

CCR5 is associated with some ligands, such as CCL3 CCL4 and CCL5 [34], that participant in the inflammatory response [35]. Increase CCL5 levels has been reported in rheumatoid arthritis (RA), and positive patient responses to methotrexate therapy have been associated with CCL5 reductions [35, 36]. Infiltrating T cells around high endothelial venules in RA synovium, and approximately 85% of synovial fluid CD3^+^CD4^+^ T cells expressed CCR5 [37]. In active systemic lupus erythematous (SLE) patients, CCR5 levels on the surface of CD4^+^ T cells were higher compared with patients in remission and healthy controls [38].

While we observed significant differences between CCR5^+/+^ and CCR5^−/−^ mice with MOG35-55-induced EAE, Tran, et al. reported very few differences [39]. These differences may be due to the fact that they injected only half the amounts of both MOG_35-55_ peptide and *Mycobacterium tuberculosis* H37RA into mice as in the present study. Zheng *et al.* reported that inhibiting CCR5 led to reduced EAE onset and development [40]. Additionally, patients with MS exhibit a higher percentage of circulating CCR5^+^ cells than controls, and an increased number of these cells is associated with disease severity [41]. This and our results indicate that CCR5 may be significant in the development of EAE.

Change of chemokine levels, including that of TNF-α, IFN-γ, MCP-1 and IL-1, are correlated with the development of EAE. The direct action of TNF-α in the pathogenesis of EAE was confirmed by peripheral administration of a neutralizing anti-murine TNF-α antibody. This treatment completely prevented T-cell infiltration into the CNS and demyelination [42]. Peripheral blood CCR5^+^ T cells secreted high levels of IFN-γ, and IFN-γ was observed in demyelinating lesions [43]. IL-1 receptor antagonists successfully prevented or suppressed EAE [44, 45]. Similarly, IL-1 receptor gene deficient mice are resistant to EAE [46]. Our data showed that TNF-α, IFN-γ and IL-1β levels in CCR5^−/−^ mice were much higher than in controls, but were lower than in CCR5^+/+^ mice.

MCP-1 is a member of the C-C chemokine family and regulates the migration and infiltration of monocytes, dendritic cells, T cells and NK cells [47]. MCP-1 levels in the CNS are dramatically increased during the relapsing phase of chronic relapsing EAE, and MCP-1 antibodies can effectively mitigate disease severity [48-50]. MCP1^−/−^ mice are also resistant to EAE and exhibit reduced CNS macrophages, neutrophille and T cells infiltration [16, 51]. In our experiments, MCP-1 was decreased in CCR5^−/−^ mice compared to CCR5^+/+^ mice. These data suggest that reduced levels of pro-inflammatory cytokines, especially IL-1 and MCP-1, could inhibit EAE development.

In MS and the EAE mice model, morphologically indistinguishable phagocytic cells accrue as a result of proliferation of resident precursors and recruitment of blood-borne progenitors, respectively [52]. Our data showed that infiltration of CD11b^+^ (monocyte), F4/80^+^ (macrophage), CD16^+^ (NK cell) and CD19^+^ (B cell) cells infiltration was decreased in CCR5^−/−^ mice spinal cord tissue compared to CCR5^+/+^ mice tissue. Previous studies regarding MS immunopathology have shown that autoreactive pro-inflammatory T cells play an important role in the propagation of CNS tissue injury. Interaction between APCs, such as macrophages and dendritic cells, and T cells lead to cytokine-mediated T cell activation and proliferation [53]. T cell-mediated immune response to various myelin antigens is a major cause of demyelination [54]. EAE is induced by immunization with myelin, myelin proteins, and myelin protein encephalitogenic epitopes, or by the passive transfer of myelin-reactive CD4 T cells [55, 56]. CD4 T cells differentiate further into T_h_1 cells which synthesize inflammatory cytokines, such as TNF-α and IFN-γ [57, 58]. Histological analysis of acute MS cases showed CD8 T cells in close proximity or attached to oligodendrocytes or demyelinated axons. Studies have also demonstrated the presence and activation of myelin-reactive and CD8^+^ T cells in the blood and cerebrospinal fluid (CSF) of MS patients [59-61]. Our data showed that CD3^+^, CD4^+^ and CD8^+^ T cell infiltration decreased in CCR5^−/−^ mice spinal cords as compared to CCR5^+/+^ mice. Our data support the findings of Simpson, Sørensen *et al.*, who detected CCR5 on lymphocytic cells, macrophages and microglial cells in actively demyelinating MS lesions [62, 63].

Other studies have also linked CCRs or CXCRs to immune cell infiltration in injury. CCR2-dificient spinal cord sections showed fewer infiltrating CD4^+^ and CD8^+^ T cells and F4/80^+^ macrophages [29], and MHV-infected CCR5−/− mice had reduced levels of infiltrating T cells present during acute disease [64]. CCR5^−/−^ mice also reportedly showed poor migration of immune cells (including NK and T cells) [65, 66]. Mohan, *et al.* found that sections of joints from anti-CXCR3 mAb-treated animals exhibited a marked decrease in synovial leukocytes infiltration [67].

The Jurkat cell, a human mature leukemic cell line, phenotypically resembles resting human T lymphocytes and has been widely used to study T cell physiology [68]. We treated Jurkat cells with concanavalin A (ConA), an antigen-independent mitogen that induces T cells proliferation [69], and DAPTA, an antagonist of CCR5-mediated chemotaxis [70]. Levels of cytokines, including TNF-α and IFN-γ, were induced by con A treatment and this effect was reversed by DAPTA, as measured by both cell viability and proliferation assays. However, IL-1 is strongly expressed by monocytes, tissue macrophages and dendritic cells [71, 72]. MCP-1 is also produced by macrophages, fibroblasts, epithelial cells and endothelial cells [73], so IL-1β and MCP-1 was rarely detected in Jurkat cells. These cytokines and chemokines were produced *in vivo* in macrophages in spinal cord tissue, and secreted IL-1β and MCP-1 might impact T cells in ways not observed in our *in vitro* cultures [47]. We concluded that CCR5 gene knockout suppresses EAE and reduces demyelination *via* regulation of immune cells and several cytokines. CCR5 is likely participating in demyelination in the spinal cord of the MS development, and could be and effective therapeutic target for treatment of MS and other neuropathological disease.

## MATERIALS AND METHODS

### Animals

CCR5^−/−^ mice (B6.129P2-*Ccr5^tm1Kuz^*/J), with full CCR5 gene coding region deletions, were obtained from The Jackson Laboratory (Bar Harbor, ME, USA). Age-matched, 8-week old CCR5^+/+^ mice (B6129PF2/J) from the same supplier were used as wild-type controls. Animals were maintained in conventional housing at 23 ± 2°C with a controlled 12 h light/dark cycle, and drinking water and rodent chow were provided throughout the experiment. All experiments were approved and carried out according to the Guidelines for the Care and Use of Animals [Animal Care Committee of Chungbuk National University, Korea (CBNUA-436-12-02)].

### EAE induction

CCR5^−/−^ and CCR5^+/+^ mice were immunized with MOG_35-55_ peptide emulsified with complete Freund's adjuvant (CFA) using Hooke kits (Hooke laboratories, EK-0115, Lawrence, MA, USA) according to the manufacturer's instructions. In brief, 0.1 mL MOG35-55/CFA emulsion was injected subcutaneously into the upper and lower back of each mouse (0.2 mL/animal) followed by intraperitoneal (i.p.) injections of 0.1 ml pertussis toxin (PTX). Twenty-four hours later, booster shots of PTX (0.1 ml/animal, i.p.) were given. Normal saline-administrated CCR5^+/+^ mice were used as controls.

### Clinical evaluation of EAE

A masked investigator examined and scored mice daily for clinical signs of neurological deficit according to the following scale: grade 0, no abnormality; grade 0.5, partial tail paralysis; grade 1, complete tail paralysis; grade 1.5, complete tail paralysis and clumsy gait; grade 2, complete tail paralysis and hind limb weakness; grade 2.5, unilateral hind limb paralysis; grade 3, complete hind limb paralysis; grade 3.5, complete hind limb paralysis and fore-limb weakness; grade 4, tetraplegia; grade 5, moribund state or death. Data were plotted as daily mean clinical score for all animals in a particular treatment group, including the scores of asymptomatic mice (score = 0) [74-76].

### Jurkat cell culture

Jurkat cells were maintained in Roswell Park Memorial Institute (RPMI) 1640 culture media supplemented with fetal bovine serum (FBS) (10%) and penicillin (100 units/ml). Cells were maintained in a humidified incubator at 37°C and 5% CO_2_, and were treated simultaneously with con A (4 μg/mL) and a CCR5 antagonist (D-Ala_1_-peptide T-NH_2_; DAPTA; Bachem Bioscience Inc., King of Prussia, PA) (10 μM) dissolved in distilled water.

### Spinal cord collection and preservation

After behavioral tests, mice were perfused with phosphate-buffered saline (PBS, pH 7.4) under inhaled diethyl ether anesthetization. Spinal cords were immediately pulled from the skull and cut. One removed spinal column was stored at −80°C; the remainder were fixed in 4% paraformaldehyde for 72 h at 4°C and transferred to 30% sucrose solutions.

### Histological analysis

Spinal cords from 30% sucrose solutions were cut into 16 ?ere cut intusing a cryostat microtome (Leica CM 1850; Leica Microsystems, Seoul, Korea) and stained with LFB/Crystal Violet (LFB, IHC World, Ellicott City, MD) and H&E for identification of intact myelin and infiltrating cells, respectively. Sections were evaluated *via* light microscopy (Olympus, Tokyo, Japan) (^×^50 or ^×^200).

### Immunofluorescence staining

After two 10-min washes in PBS (pH 7.4), endogenous peroxidase activity was quenched by incubating prepared spinal cord sections in 3% hydrogen peroxide in PBS for 20 min, followed by an additional two 10-min washes in PBS. Sections were blocked for 1 h in 5% bovine serum albumin (BSA) and incubated overnight at 4°C with a mouse polyclonal antibody against CNPase, MBP, NG2, O4 (1:200; Millipore, Billerica, MA, USA), CD3 or glial fibrillary acidic protein (GFAP) (1:200; Santa Cruz Biotechnology, Inc., Santa Cruz, CA, USA), a rat polyclonal antibody against F4/80 (1:100; Santa Cruz Biotechnology, Inc., Santa Cruz, CA, USA), CD4 (1:100, BD Biosciences, Franklin Lakes, NJ, USA), CD8b, CD11b or CD16/CD32 (1:100, eBioscince, San Diego, USA), or a goat polyclonal antibody against ionized calcium-binding adapter molecule 1 (IBA-1) (1:300; Abcam, Inc., Cambridge, MA, USA). Sections were then washed three times (10 min each) in PBS and incubated for 1-2 h at room temperature with a secondary antibody conjugated to Alexa Fluor 488 or 568 (Invitrogen-Molecular Probes, Carlsbad, CA, USA). Sections were then washed three times (10 min each) in PBS, incubated for 30 sec at room temperature in the dark for DAPI staining and mounted with Vecta mountTM AQ (Vecta Laboratories, Burlingame, CA). IF images were acquired using an inverted Zeiss Axiovert 200 M fluorescent microscope (Carl Zeiss, Thornwood, NY, USA) (X100 or X200).

### Measurement of cytokines

Protein was extracted from spinal cord tissues (lumbar regions) using protein extraction buffer (PRO-PREP^TM^, Intron Biotechnology, Korea) with protease inhibitors, incubated on ice for 2 h and centrifuged at 13,000 g for 15 min at 4°C. TNF-α, IFN-γ, IL-1β and MCP-1 levels were determined using ELISA Kits (R&D Systems, Minneapolis, MN, USA). In brief, 100 μg/μl of sample was added into a precoated plate and incubated 2 h at RT. After washing each well of the precoated plate with a washing buffer, 100 μl of labeled antibody solution was added, and the mixture was incubated for 2 h at RT. After washing, chromogen was added, and the mixture was incubated for 30 min at RT in the dark. The resulting color was assayed at 450 nm using a microplate absorbance reader (Sunrise™, Tecan, Switzerland) after adding stop solution within 30 minutes.

### Cell viability assay

Jurkat cells were plated at a density of 1 × 10^4^ cells/well in 96-well plates in 200 μL medium with DAPTA (10 μM). After 2 hr, Jurkat cells were stimulated by con A (4 μg/mL) in 200 μL medium for 12 h. The cells were added to 30 μL MTT [3-(4,5-dimethylthiazol-2-yl)-2,5-diphenyl-tetrazoliumbromide] solution (final concentration of 5 mg/mL) (Sigma, St. Louis, MO, USA). After 3 h, MTT solution was removed, and the cells were added to dimethyl sulfoxide (DMSO) in 200 μL for 30 min. Finally, the resulting color was assayed at 540 nm using a microplate absorbance reader (Sunrise™, Tecan, Switzerland).

### BrdU assay

Jurkat cells were plated at 1 × 10^4^ cells/well in 96-well plates in 200 μL medium with DAPTA (10 μM). After 2 hr, Jurkat cells were stimulated by con A (4 μg/mL) in 200 μL medium for 12 h. Detection of BrdU incorporation was performed by ELISA (BrdU Cell Proliferation Assay Kit, Cell Signaling Technology, Danvers, MA, USA) according to the manufacturer's instructions.

### Quantitative real-time PCR (qPCR)

For mRNA quantification, total RNA was extracted using the easy-BLUR^TM^ total RNA extraction kit (iNtRON Biotech, Daejeon, Korea). cDNA was synthesized using High Capacity cDNA Reverse Transcription Kits (Applied Biosystems, Foster city, CA) according to the manufacturer's instructions. Briefly, 2 μg of total RNA was used for cDNA preparation. Quantitative real-time PCR was performed using the Brilliatn III Ultra-Fast Green QPCR Master Mix (Agilent Technologies, Waldbronn, Germany) specific for β-actin (N1080, Bioneer, Daejeon, Korea), TNF-α (N-1072, Bioneer, Daejeon, Korea), IFN-γ (N-1055, Bioneer, Daejeon, Korea), IL-1β (N-1058, Bioneer, Daejeon, Korea) and MCP-1 (5′-CCT TCA TTC CCC AAG GGC TC-3′ and 5′-GGT TTG CTT GTC CAG GTG GT-3′). All reverse transcription reactions were run in a StepOnePlus Real-Time PCR System (Applied Biosystems, Foster city, CA) using the universal cycling parameters (3 min 95°C, 40 cycles of 5 s 95°C, 12 s 60°C). Results were normalized to β-actin and quantified relative to expression in control samples. For relative quantification calculation, the 2^−Δ Δ*C*T^ formula was used, where: -Δ Δ CT = (C_T,target_ - C_T,beta-actin_) experimental sample - (C_T,target_ - C_T,beta-actin_) control sample.

### Statistical analysis

Statistical analysis were carried out using analysis of variance (ANOVA) for repeated measures followed by Dunnette's post-hoc analysis using GraphPad Prism 5 software (Version 5.01, GraphPad software, Inc., La Jolla, USA). Immune cell counts were determined using the ImageJ.

## SUPPLEMENTARY MATERIAL FIGURES


