# Supplementary material for: CCR5 knockout suppresses experimental autoimmune encephalomyelitis in C57BL/6 mice
Source: Oncotarget. 2016 Mar 15;7(13):15382–93. doi: 10.18632/oncotarget.8097 (PMC4941248; doi:10.18632/oncotarget.8097)
Supplement: Supplementary file 1 [file oncotarget-07-15382-s001.pdf]

CCR5 knockout suppresses experimental autoimmune encephalomyelitis in C57BL/6 mice

Supplementary Material

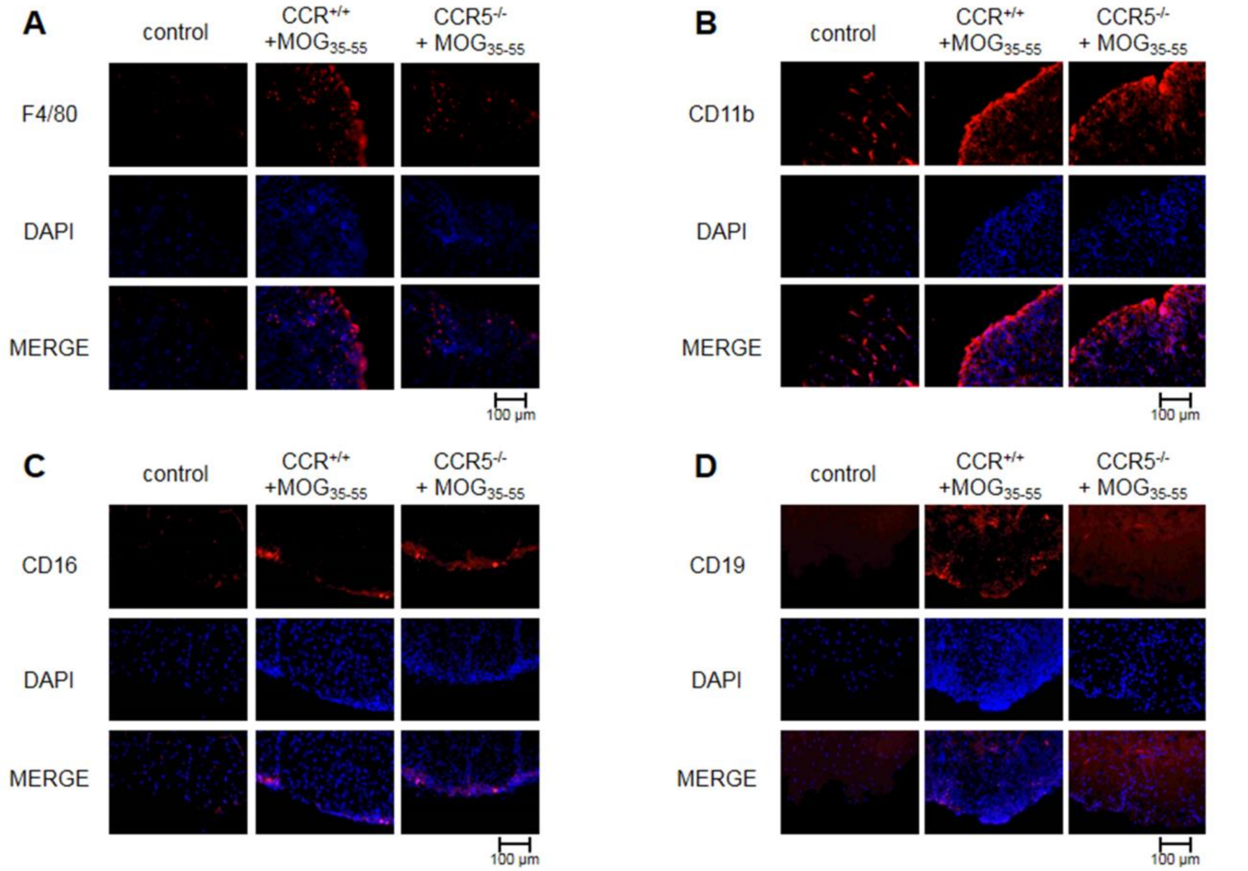

Supplementary Figure 1: Immune cell infiltration as shown by IF staining in 16 μm-thick mouse spinal cord sections. F4/80, CD11b$^+$, CD16$^+$ and CD19$^+$ cells did not infiltrate control mice spinal cord tissues, but did infiltrate MOG_{35-55}-induced mouse spinal cords **A.–D.**

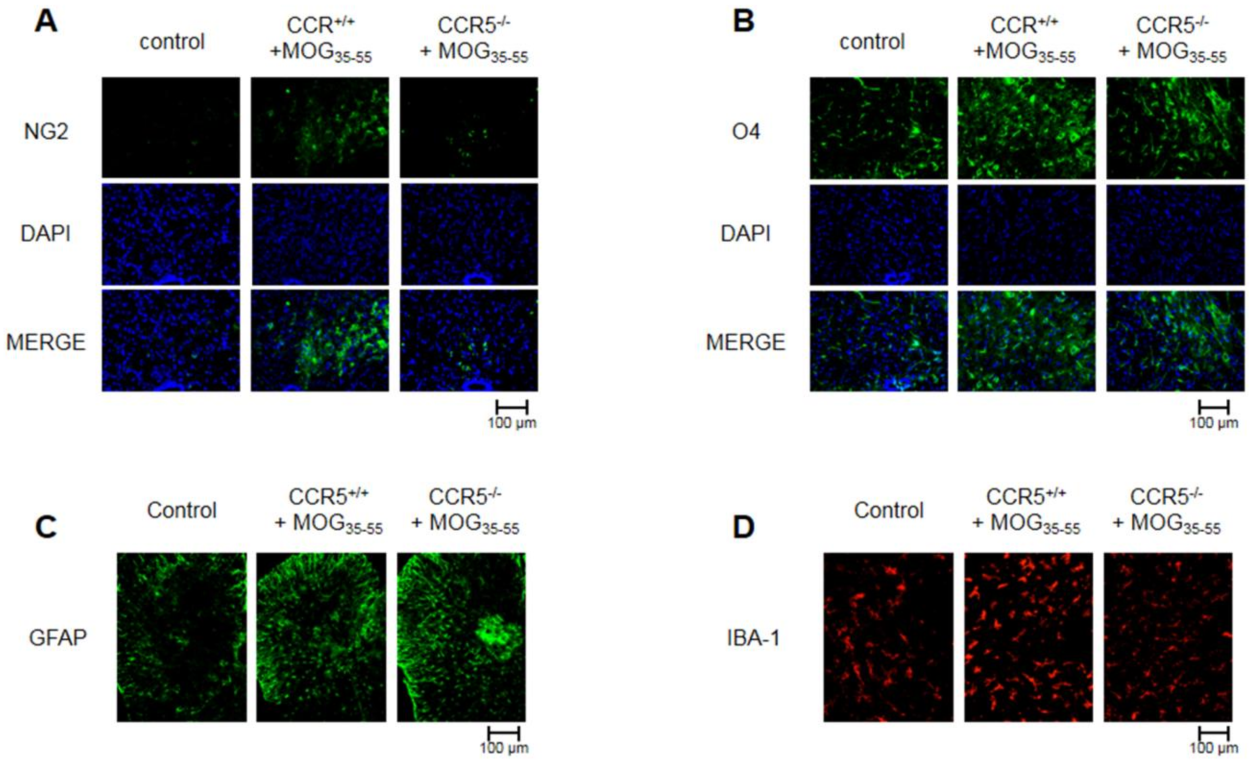

Supplementary Figure 2: Oligodendrocyte progenitor cell marker expression as shown by IF staining in 16 µm-thick mouse spinal cord sections. NG2 (oligodendrocyte precursor maker) and O4 (oligodendrocyte maker) expression increased in MOG$_{35-55}$-induced CCR5$^{+/+}$ mouse spinal cords compared to CCR5$^{-/-}$ mice **A. & B.** GFAP (microglial marker) and IBA-1 (astrocyte marker) expression increased in MOG$_{35-55}$-induced CCR5$^{+/+}$ mouse spinal cords compared to CCR5$^{-/-}$ mice **C. & D.**
